# Supplementary material for: Shared structural features of Miro binding control mitochondrial homeostasis
Source: EMBO J. 2024 Jan 24;43(4):6. doi: 10.1038/s44318-024-00028-1 (PMC10897228; doi:10.1038/s44318-024-00028-1)
Supplement: Supplementary file 1 — Appendix [file 44318_2024_28_MOESM1_ESM.pdf]

Appendix

Contents

Appendix Figure S1 – Page 2

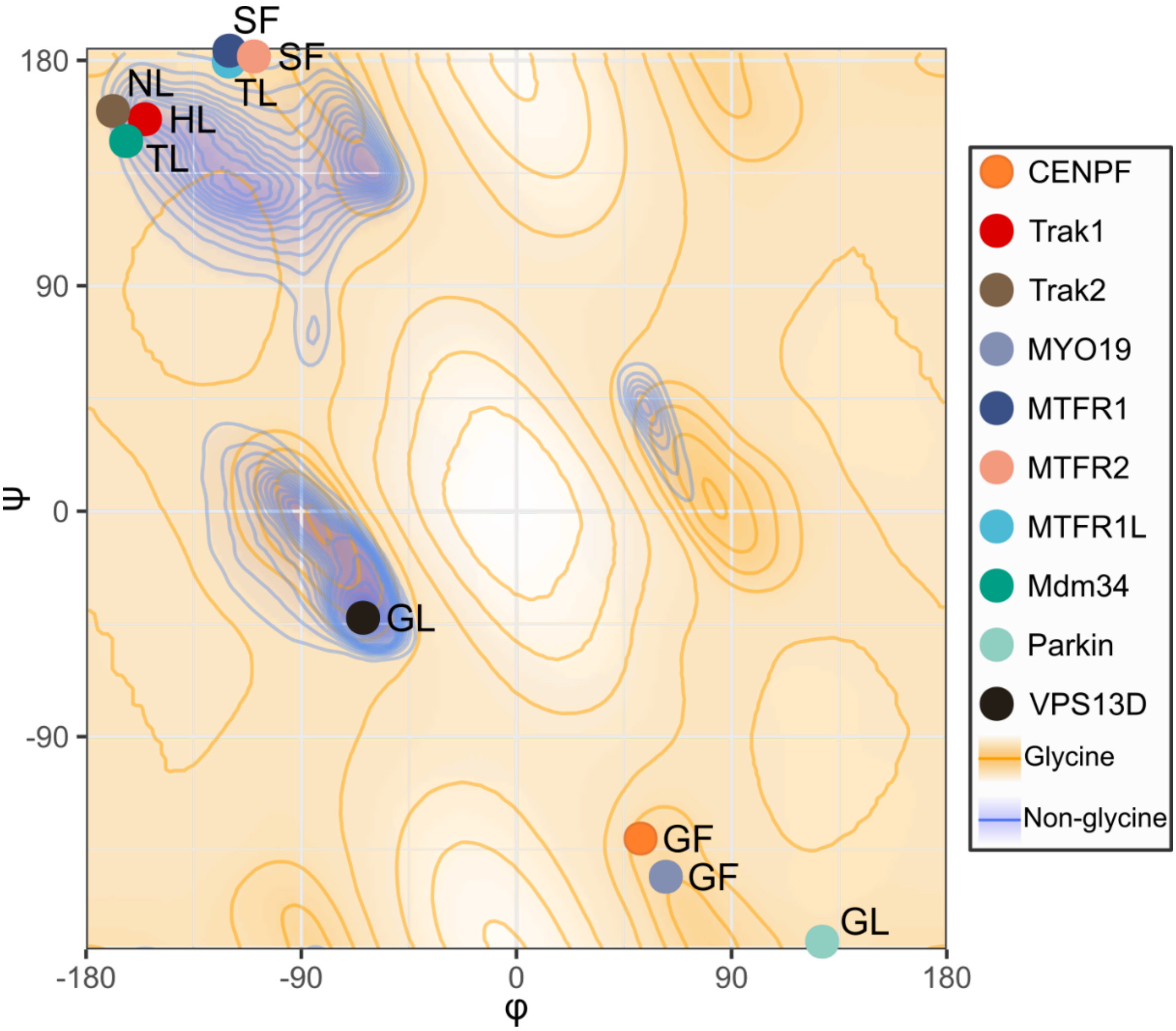

Appendix Figure S1. Angle requirements of different Miro-binding motifs. (A) Ramachandran plot depicting the bond angles of the residue preceding the ELF-binding F or L from different Miro-binding motifs. Blue and Orange depict the areas tolerating non-glycine and glycine residues respectively.
