# Supplementary figures and images for: Shared structural features of Miro binding control mitochondrial homeostasis
Source: EMBO J. 2024 Jan 24;43(4):6. doi: 10.1038/s44318-024-00028-1 (PMC10897228; doi:10.1038/s44318-024-00028-1)

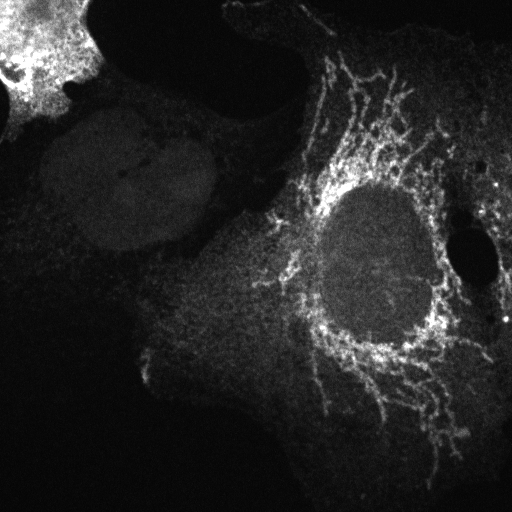

Supplement: Supplementary file 4 — Source Data Fig. 2 [file 44318_2024_28_MOESM4_ESM.zip › Figure 2/2F/C1-GFP-Myo19 C2-Tom20-568 C3-flag-Miro1-647.tif]

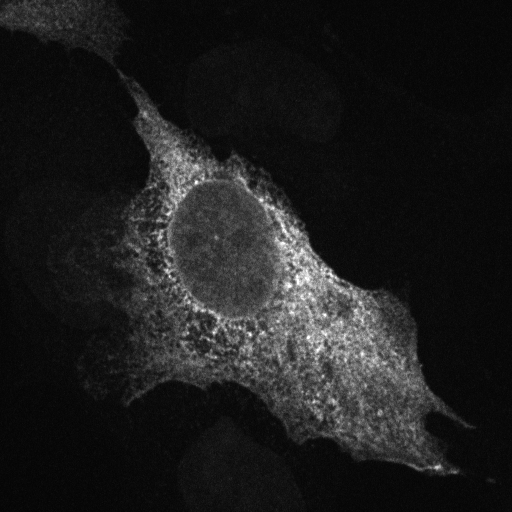

Supplement: Supplementary file 4 — Source Data Fig. 2 [file 44318_2024_28_MOESM4_ESM.zip › Figure 2/2F/C1-GFP-Myo19-F948A C2-Tom20-568 C3-flag-Miro1-647.tif]

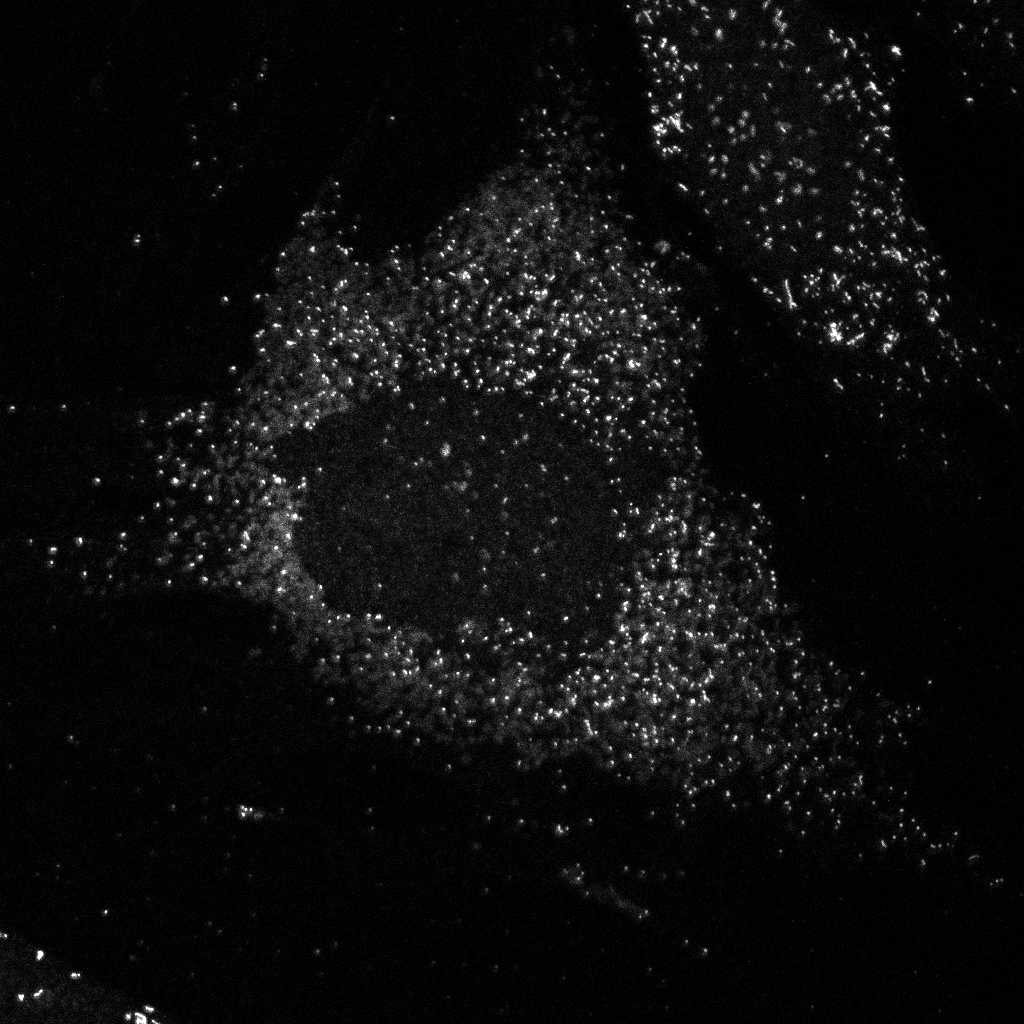

Supplement: Supplementary file 5 — Source Data Fig. 3 [file 44318_2024_28_MOESM5_ESM.zip › Figure 3/3B/MTFR1 in DKO_C1-IGNORE C2-IGNORE C3-MtdsRed C4-Mtfr1-myc.tif]

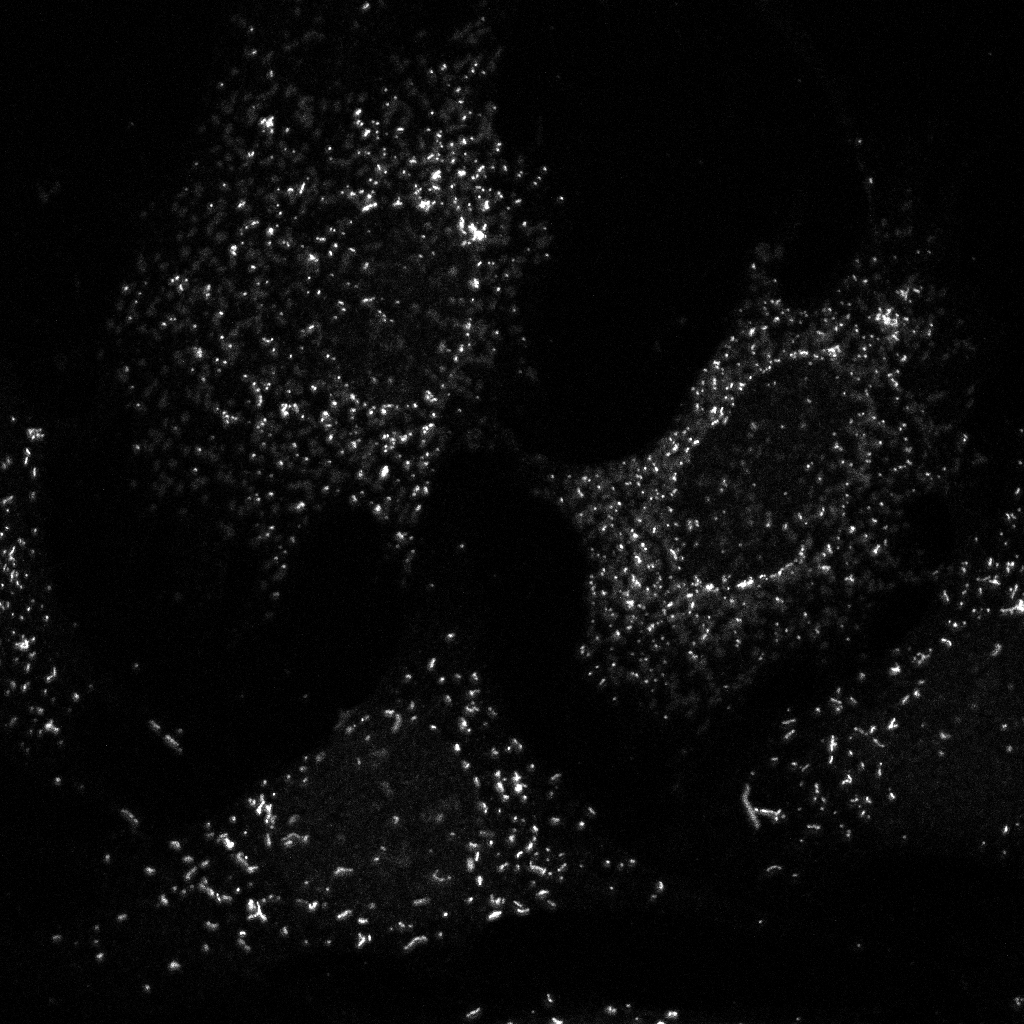

Supplement: Supplementary file 5 — Source Data Fig. 3 [file 44318_2024_28_MOESM5_ESM.zip › Figure 3/3B/MTFR1 in WT_C1-IGNORE C2-IGNORE C3-MtdsRed C4-Mtfr1-myc.tif]

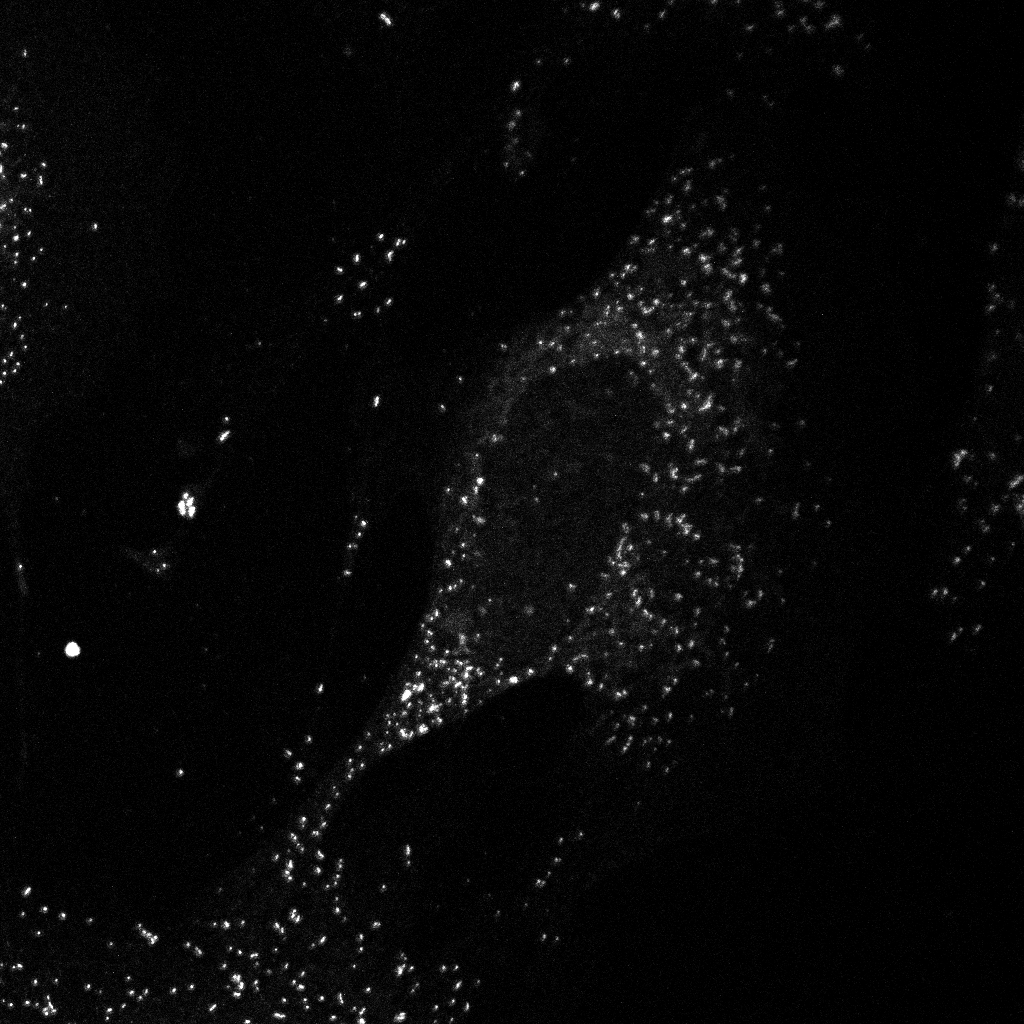

Supplement: Supplementary file 5 — Source Data Fig. 3 [file 44318_2024_28_MOESM5_ESM.zip › Figure 3/3B/MTFR1L in DKO_C1-IGNORE C2-IGNORE C3-MtdsRed C4-Mtfr1L-myc.tif]

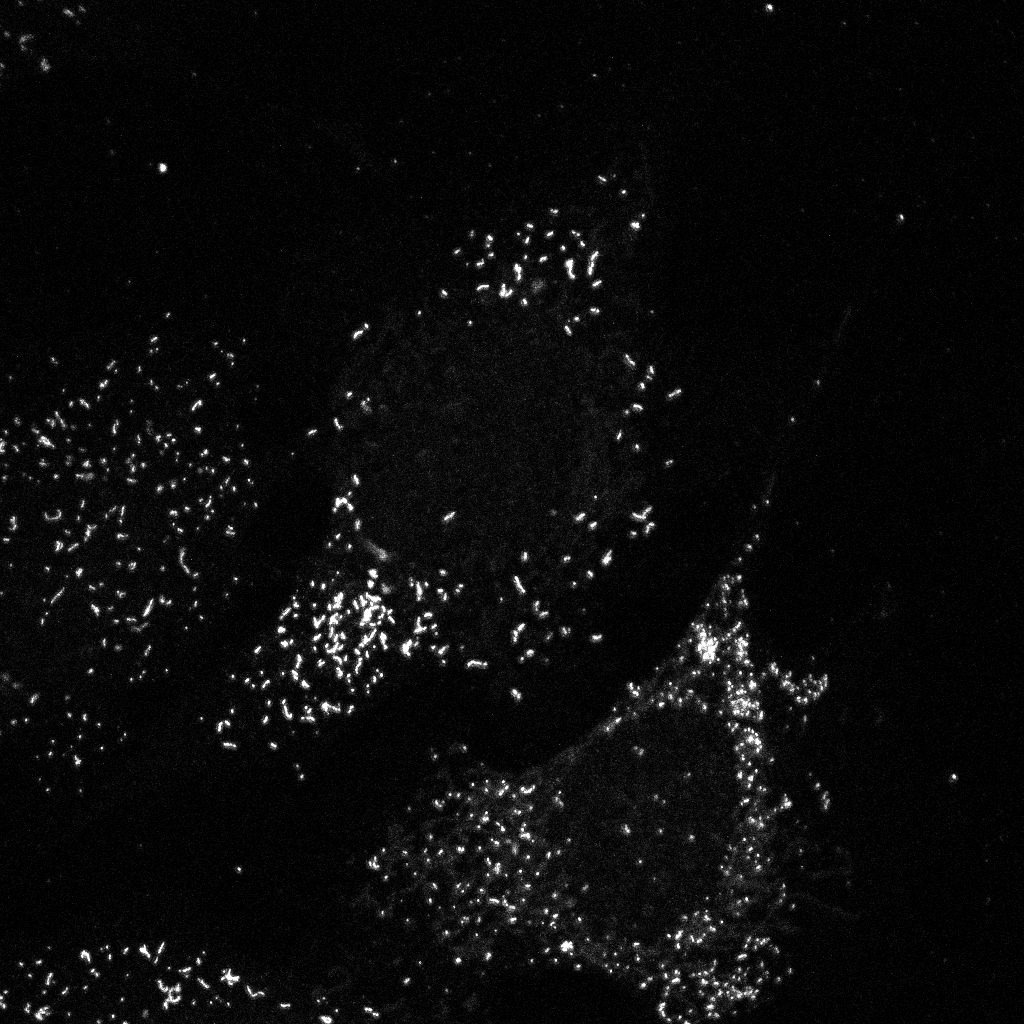

Supplement: Supplementary file 5 — Source Data Fig. 3 [file 44318_2024_28_MOESM5_ESM.zip › Figure 3/3B/MTFR1L in WT_C1-IGNORE C2-IGNORE C3-MtdsRed C4-Mtfr1L-myc.tif]

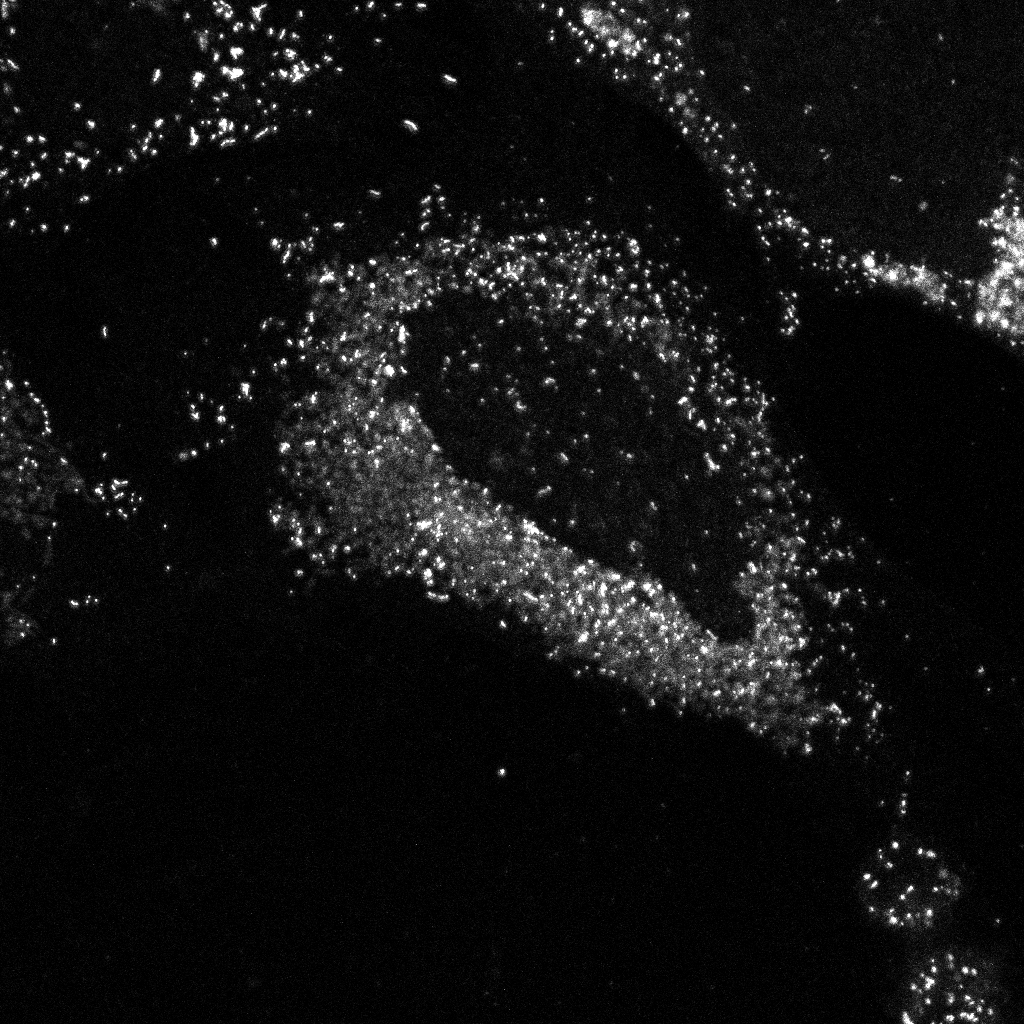

Supplement: Supplementary file 5 — Source Data Fig. 3 [file 44318_2024_28_MOESM5_ESM.zip › Figure 3/3B/MTFR2 in DKO_C1-IGNORE C2-IGNORE C3-MtdsRed C4-Mtfr2-myc.tif]

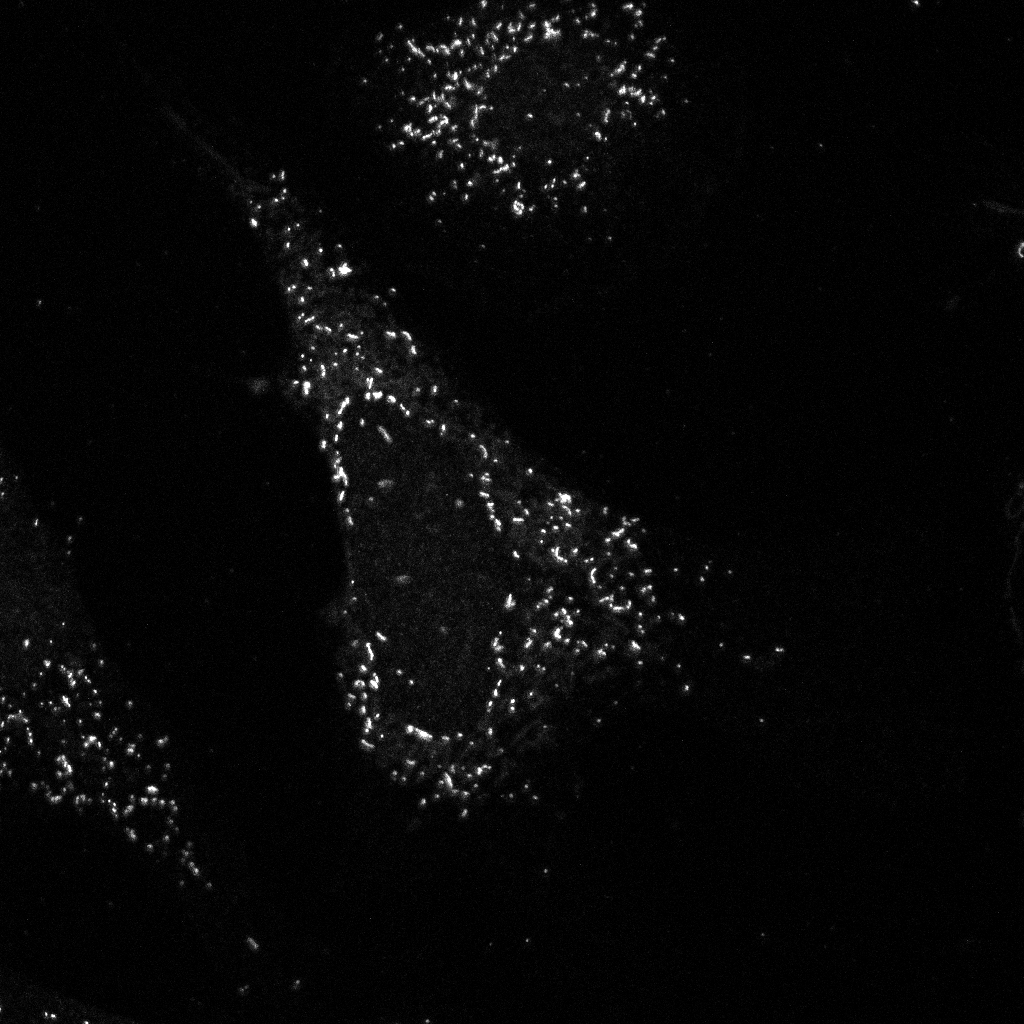

Supplement: Supplementary file 5 — Source Data Fig. 3 [file 44318_2024_28_MOESM5_ESM.zip › Figure 3/3B/MTFR2 in WT_C1-IGNORE C2-IGNORE C3-MtdsRed C4-Mtfr2-myc.tif]

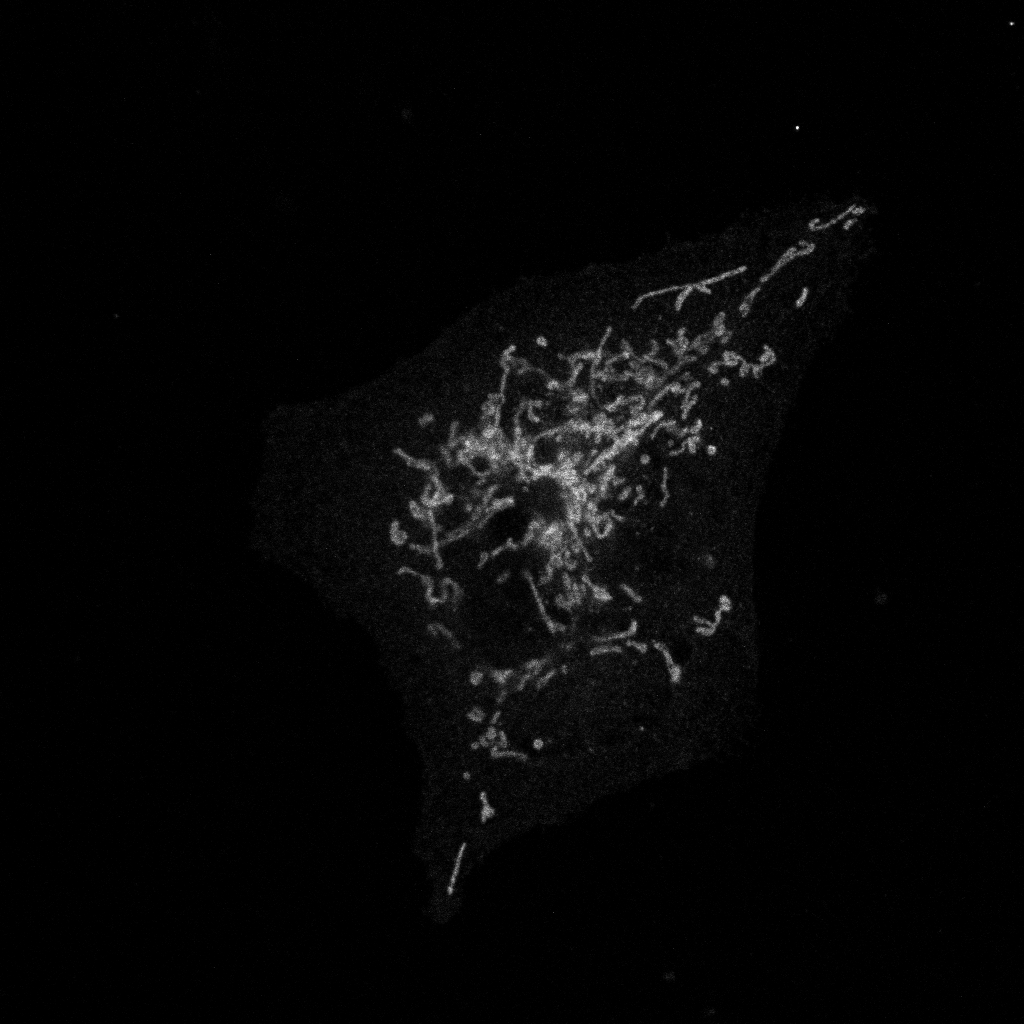

Supplement: Supplementary file 5 — Source Data Fig. 3 [file 44318_2024_28_MOESM5_ESM.zip › Figure 3/3D/Mtfr1l + Miro1_C1-IGNORE C2-mtDsRed C3 MTFR1L-GFP.tif]

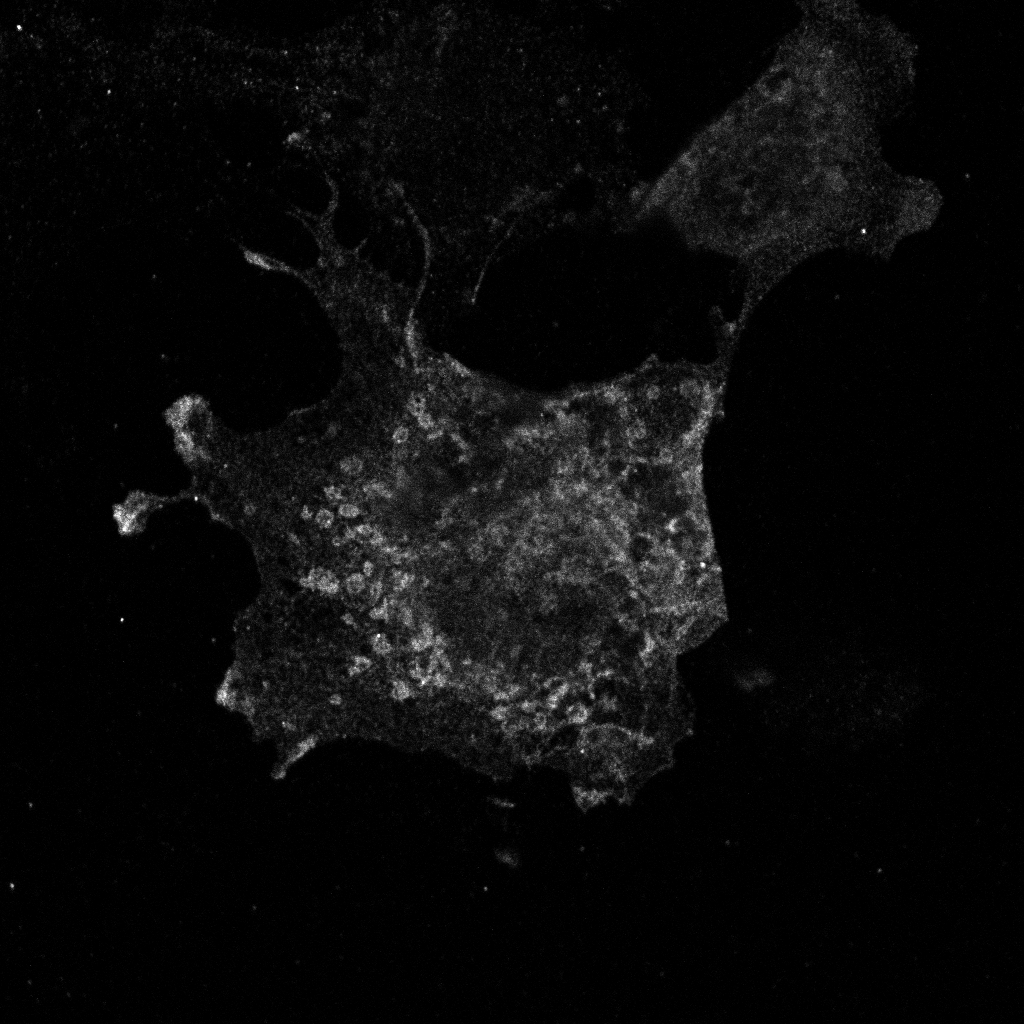

Supplement: Supplementary file 5 — Source Data Fig. 3 [file 44318_2024_28_MOESM5_ESM.zip › Figure 3/3D/Mtfr1l_C1-IGNORE C2-mtDsRed C3 MTFR1L-GFP.tif]

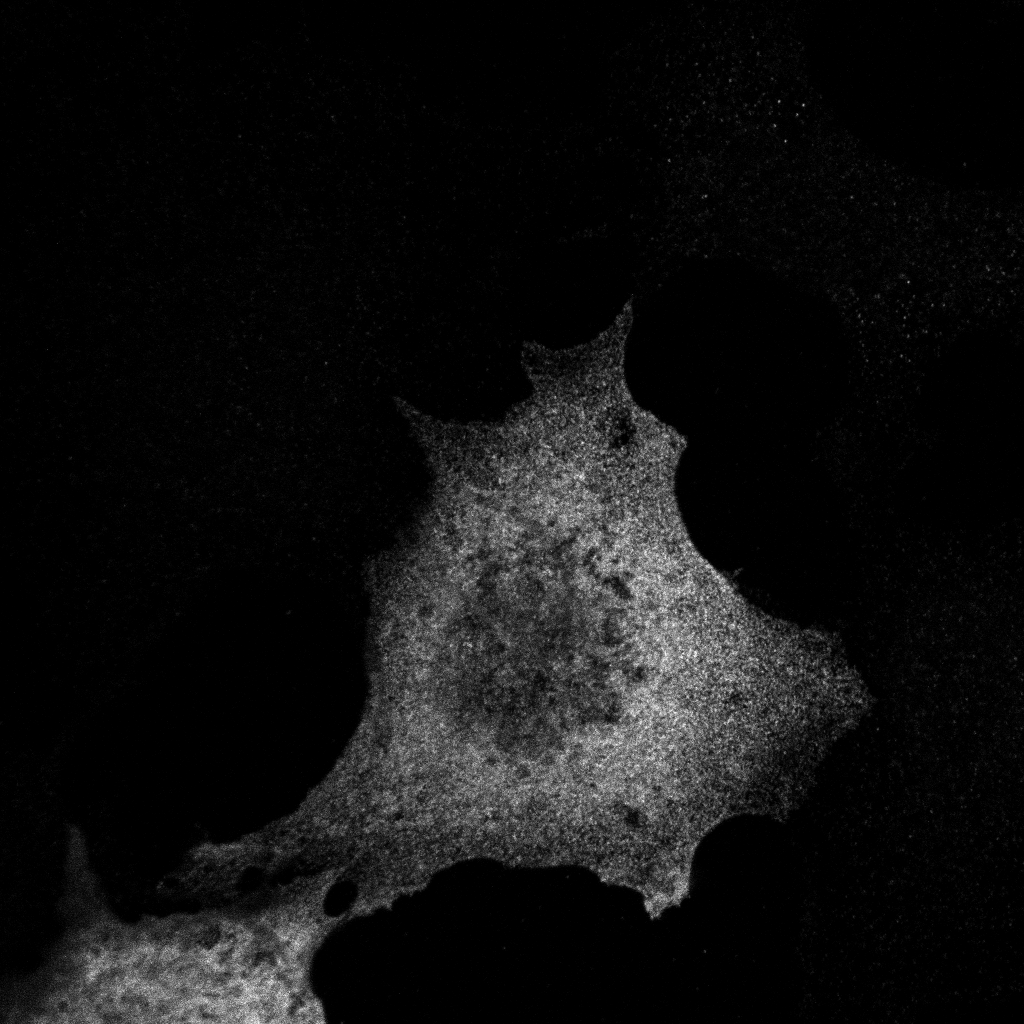

Supplement: Supplementary file 5 — Source Data Fig. 3 [file 44318_2024_28_MOESM5_ESM.zip › Figure 3/3D/Mtfr1l-L62A + Miro1_C1-IGNORE C2-mtDsRed C3 MTFR1L-GFP.tif]

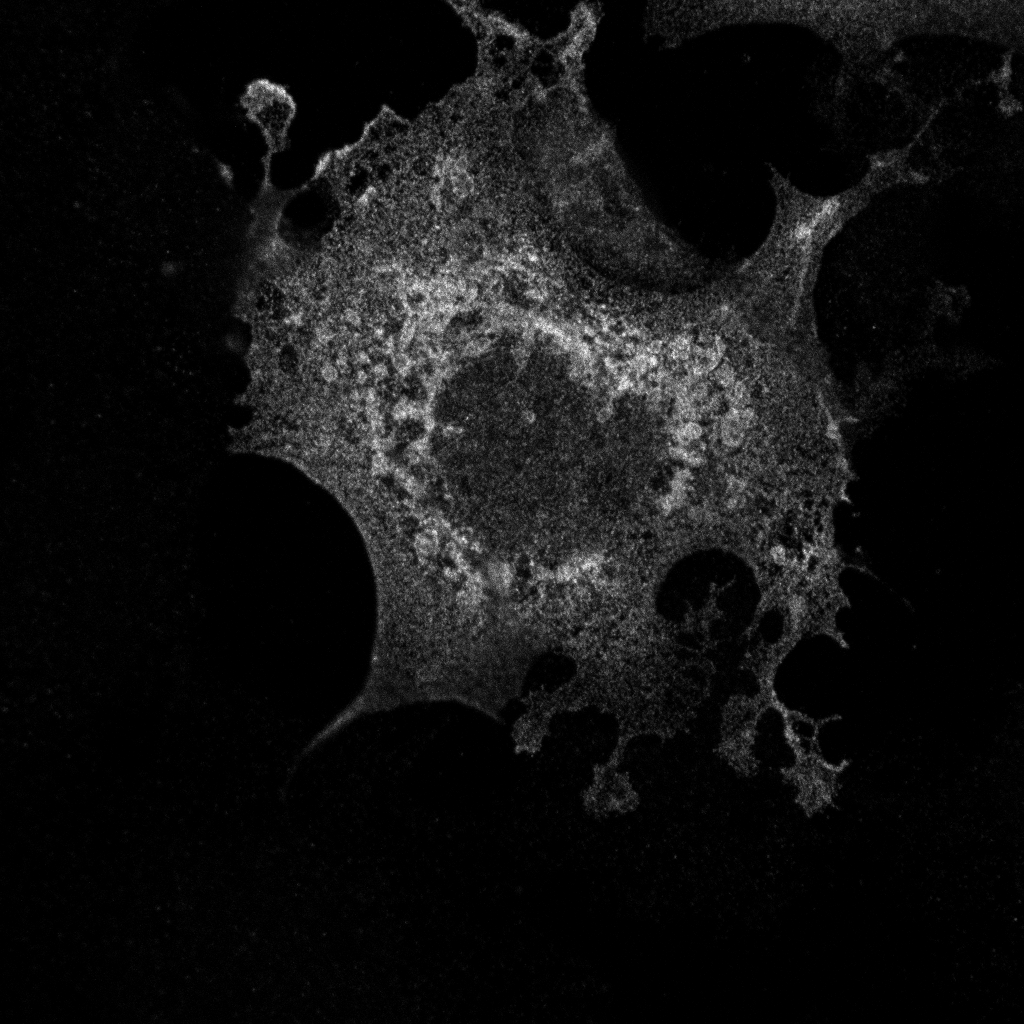

Supplement: Supplementary file 5 — Source Data Fig. 3 [file 44318_2024_28_MOESM5_ESM.zip › Figure 3/3D/Mtfr1l-L62A_C1-IGNORE C2-mtDsRed C3 MTFR1L-GFP.tif]

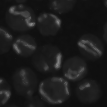

Supplement: Supplementary file 6 — Source Data Fig. 4 [file 44318_2024_28_MOESM6_ESM.zip › Figure 4/4C/Mdm34-L248A C1-Gem1^GFP C2-Mdm34-mCherry C3-brightfield.tif]

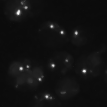

Supplement: Supplementary file 6 — Source Data Fig. 4 [file 44318_2024_28_MOESM6_ESM.zip › Figure 4/4C/WT C1-Gem1^GFP C2-Mdm34-mCherry C3-brightfield.tif]

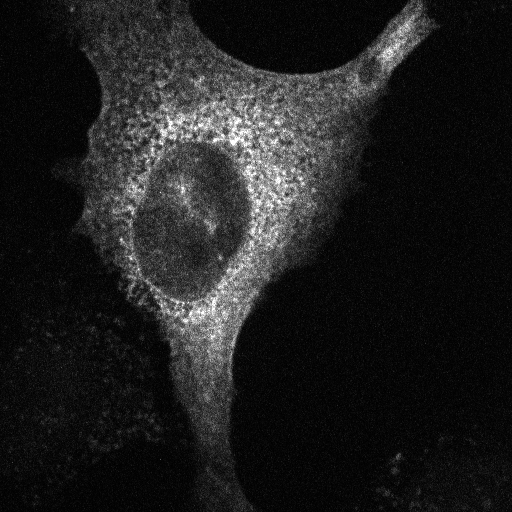

Supplement: Supplementary file 7 — Source Data Fig. 5 [file 44318_2024_28_MOESM7_ESM.zip › Figure 5/5C/C1- VPS13D-L2554A^GFP C2-Miro1-647.tif]

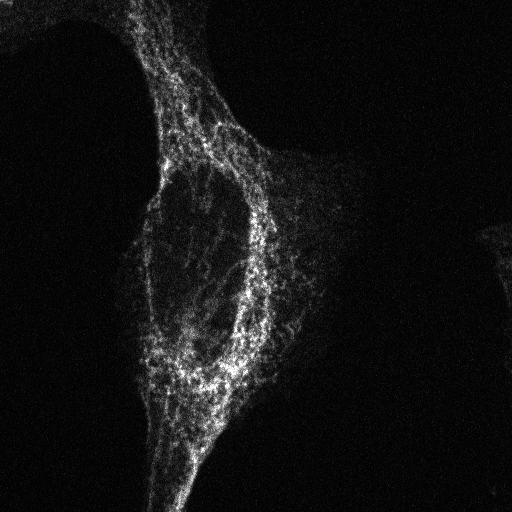

Supplement: Supplementary file 7 — Source Data Fig. 5 [file 44318_2024_28_MOESM7_ESM.zip › Figure 5/5C/C1-VPS13D^GFP C2-Miro1-647.tif]

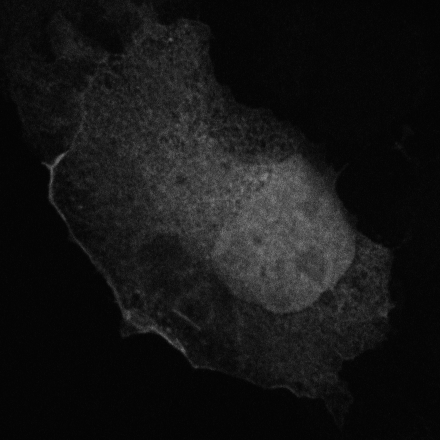

Supplement: Supplementary file 7 — Source Data Fig. 5 [file 44318_2024_28_MOESM7_ESM.zip › Figure 5/5G/C1-YFP-Parkin C2-Tom20-568 C3-empty.tif]

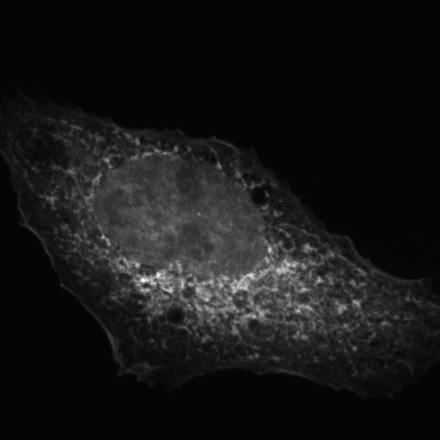

Supplement: Supplementary file 7 — Source Data Fig. 5 [file 44318_2024_28_MOESM7_ESM.zip › Figure 5/5G/C1-YFP-Parkin C2-Tom20-568 C3-myc-Miro1-647.tif]

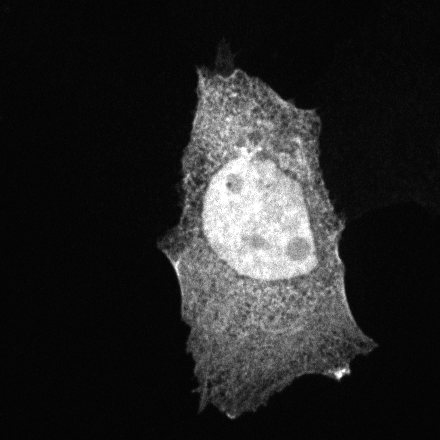

Supplement: Supplementary file 7 — Source Data Fig. 5 [file 44318_2024_28_MOESM7_ESM.zip › Figure 5/5G/C1-YFP-Parkin-L119A C2-Tom20-568 C3-empty.tif]

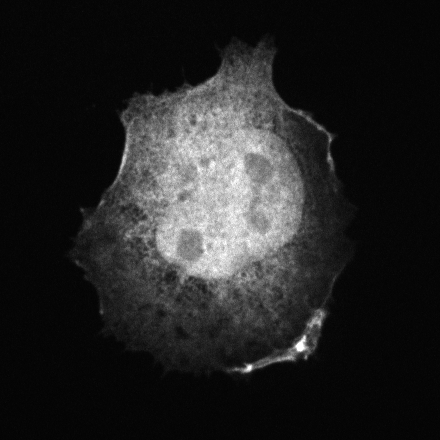

Supplement: Supplementary file 7 — Source Data Fig. 5 [file 44318_2024_28_MOESM7_ESM.zip › Figure 5/5G/C1-YFP-Parkin-L119A C2-Tom20-568 C3-mycMiro1-647.tif]
