# Supplementary material for: Shared structural features of Miro binding control mitochondrial homeostasis
Source: EMBO J. 2024 Jan 24;43(4):6. doi: 10.1038/s44318-024-00028-1 (PMC10897228; doi:10.1038/s44318-024-00028-1)
Supplement: Supplementary file 9 — Expanded View Figures [file 44318_2024_28_MOESM9_ESM.pdf]

Expanded View Figures

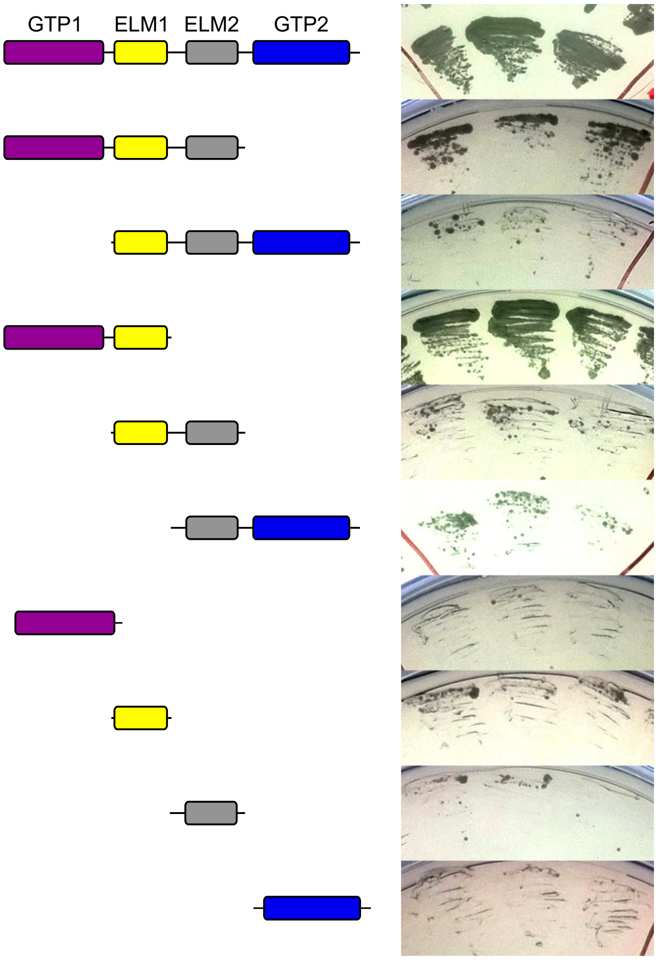

Figure EV1. GTPase1 and ELM1 of MIRO1 are necessary and sufficient for CENPF binding.

Representative yeast two-hybrid of CENPF-2819-3114 (bait) with MIRO1 truncations (prey). Each streak is from an independently generated strain. Growth of the second fragment was less robust than that of the first and fourth fragments because the expression of this fragment was poor.

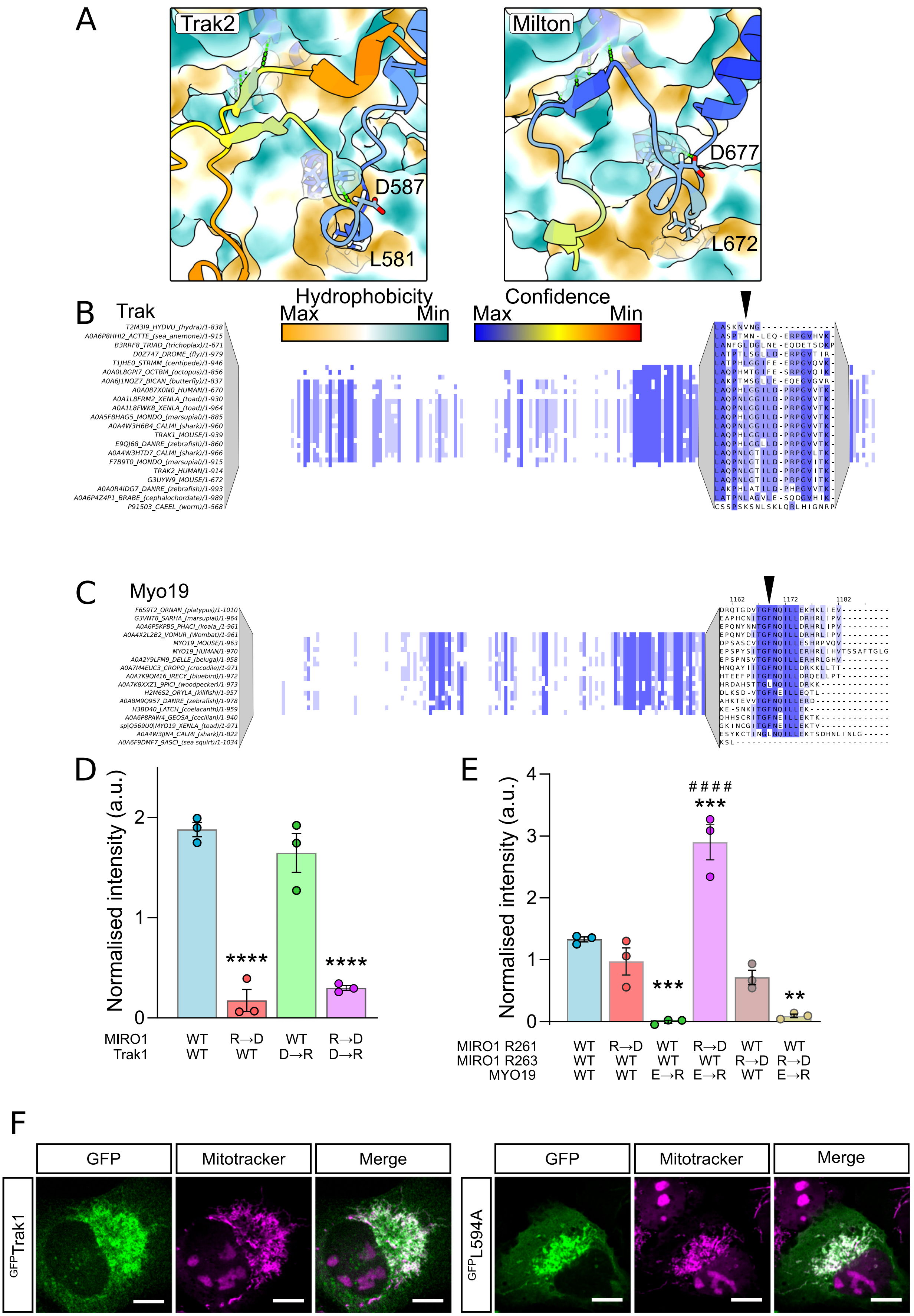
A
Trak2
D587
L581
Milton
D677
L672
B
Trak
Hydrophobicity
Max
Min
Confidence
Max
Min
C
Myo19
D
Normalised intensity (a.u.)
MIRO1 WT R→D WT R→D
Trak1 WT WT D→R D→R
E
MIRO1 R261 WT R→D WT R→D WT WT
MIRO1 R263 WT WT WT WT R→D R→D
MYO19 WT WT E→R E→R WT E→R
F
GFP
Mitotracker
Merge
GFPTrak1
GFPL594A

 © The Author(s)

Figure EV2. Conserved features in Trak1 and MYO19 that interact with MIRO1.

(**A**) AlphaFold2 multimer predictions of human Trak2 with MIRO1 (surface) or *Drosophila melanogaster* Miro (surface) and Milton (Trak orthologue). Color coding as in Fig. 1D. (**B**) Sequence alignment of Trak orthologues around the Miro-binding motif. (**C**) Sequence alignment of MYO19 orthologues around the Miro-binding motif. (**D**) Quantification of fluorescence yeast two-hybrid of wild-type and charge swapped mutants of MIRO1 (prey) and mouse Trak1-577-620 (bait). R → D and D → R are MIRO1-R263D and Trak1-D599R, respectively, $n = 3$. (**E**) Quantification of fluorescence yeast two-hybrid of wild-type and charge swapped mutants of MIRO1 (prey) and MYO19-919-970 (bait). R → D and E → R are MIRO1-R261D/MIRO1-R263D and MYO19-E954R, respectively, $n = 3$. (**F**) Representative images of wild-type and L594A mouse ^{GFP}Trak1 (green) in U2OS cells. Mitochondria are stained with Mitotracker-Orange (magenta). Scale bars represents 10 µm. Data information: D and E statistical significance was calculated by one-way ANOVA with Tukey post hoc test. **, *** and **** denotes $P < 0.01$, 0.001 and 0.0001 in comparison to WT conditions. $^{####}$ is $P < 0.0001$ in comparison to WT-MIRO1 + MYO19-E → R.

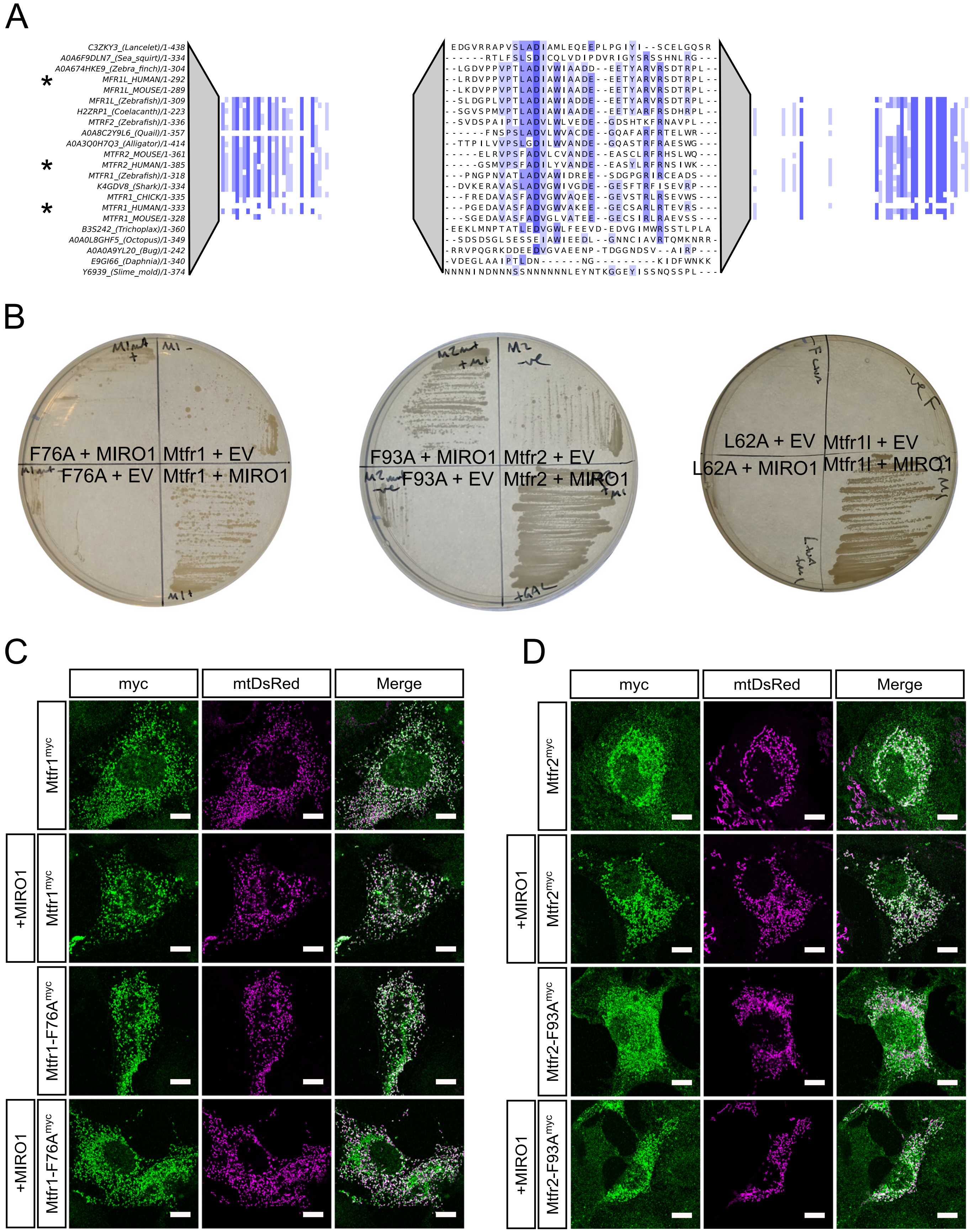
A
B
F76A + MIRO1
Mtfr1 + EV
F76A + EV
Mtfr1 + MIRO1
F93A + MIRO1
Mtfr2 + EV
F93A + EV
Mtfr2 + MIRO1
L62A + EV
Mtfr1l + EV
L62A + MIRO1
Mtfr1l + MIRO1
C
myc
mtDsRed
Merge
+MIRO1
D

 © The Author(s)

Figure EV3. Mtfr1/2/1L interact with MIRO1 via conserved motif.

(**A**) Sequence alignment of sequences of MTFR1, MTFR2 and MTFR1L surrounding the Miro-binding motif. (**B**) Yeast two-hybrid growth assay of MIRO1 (prey) with wild-type or point mutants of full-length Mtfr1, Mtfr2 and Mtfr1l. EV means empty vector control. (**C**) Representative images of U2OS Cos7 cells expressing either wild-type or F76A point mutated Mtfr1 (green) both with or without ^{GFP}MIRO1 overexpression. Mitochondria are stained with mtDsRed (magenta). (**D**) Representative images of U2OS Cos7 cells expressing either wild-type or F93A point mutated Mtfr2 (green) both with and without ^{GFP}MIRO1 overexpression. Mitochondria are stained with mtDsRed (magenta). Scale bars depict 10 μm.

© The Author(s)

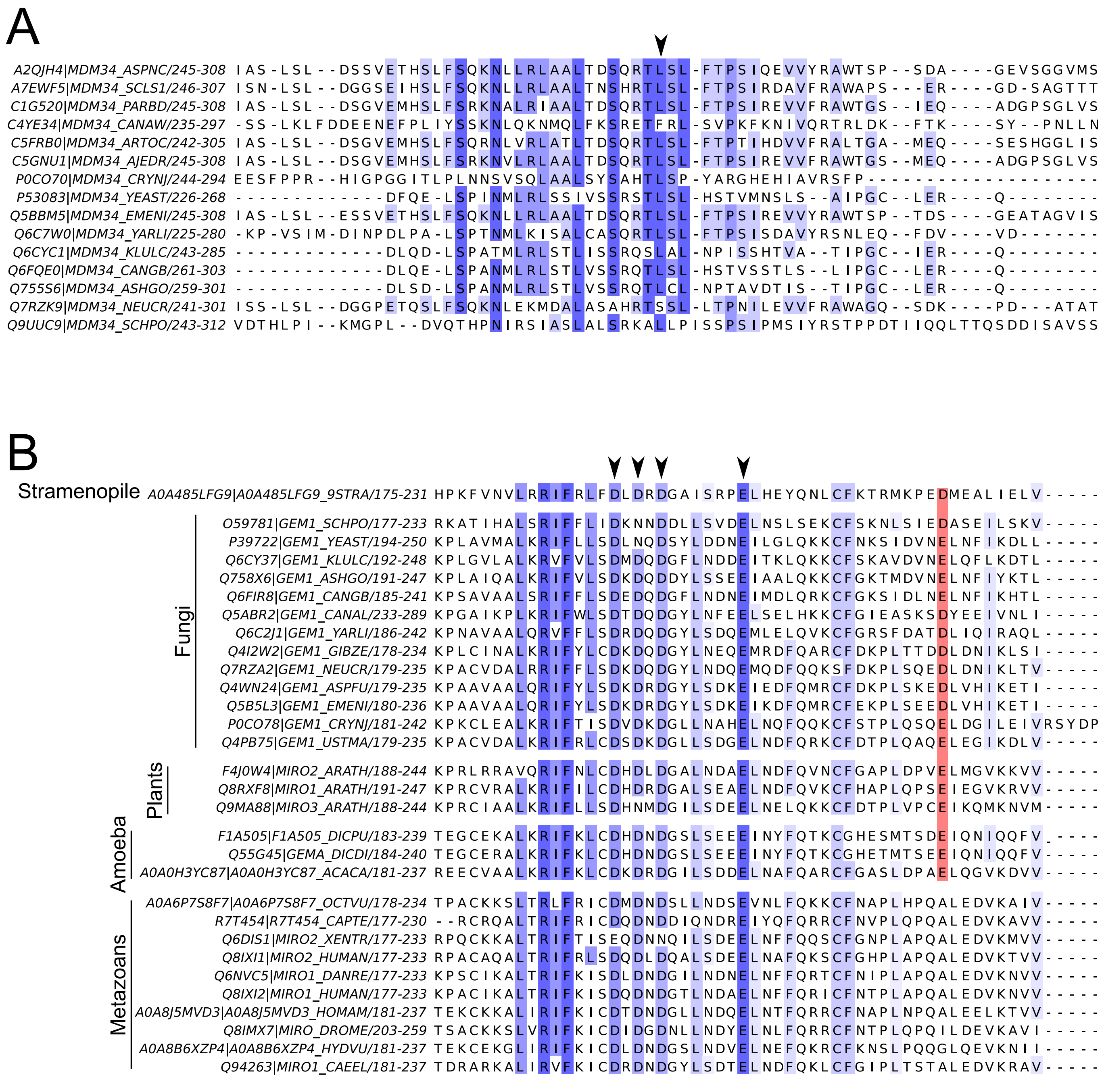

Figure EV4. Conservation of Gem1 and Mdm34.

(**A**) Sequence alignment of Mdm34. Arrowheads indicate the position of the Leucine inserted in the ELF. (**B**) Sequence alignments of Miro orthologues in fungi, plants, amoeba, and metazoans. Arrowheads indicate the acidic residues coordinating Ca^{2+} in the EF hand. Red highlights key acidic residue not found in metazoans that is likely required for Mdm34 binding.

 © The Author(s)

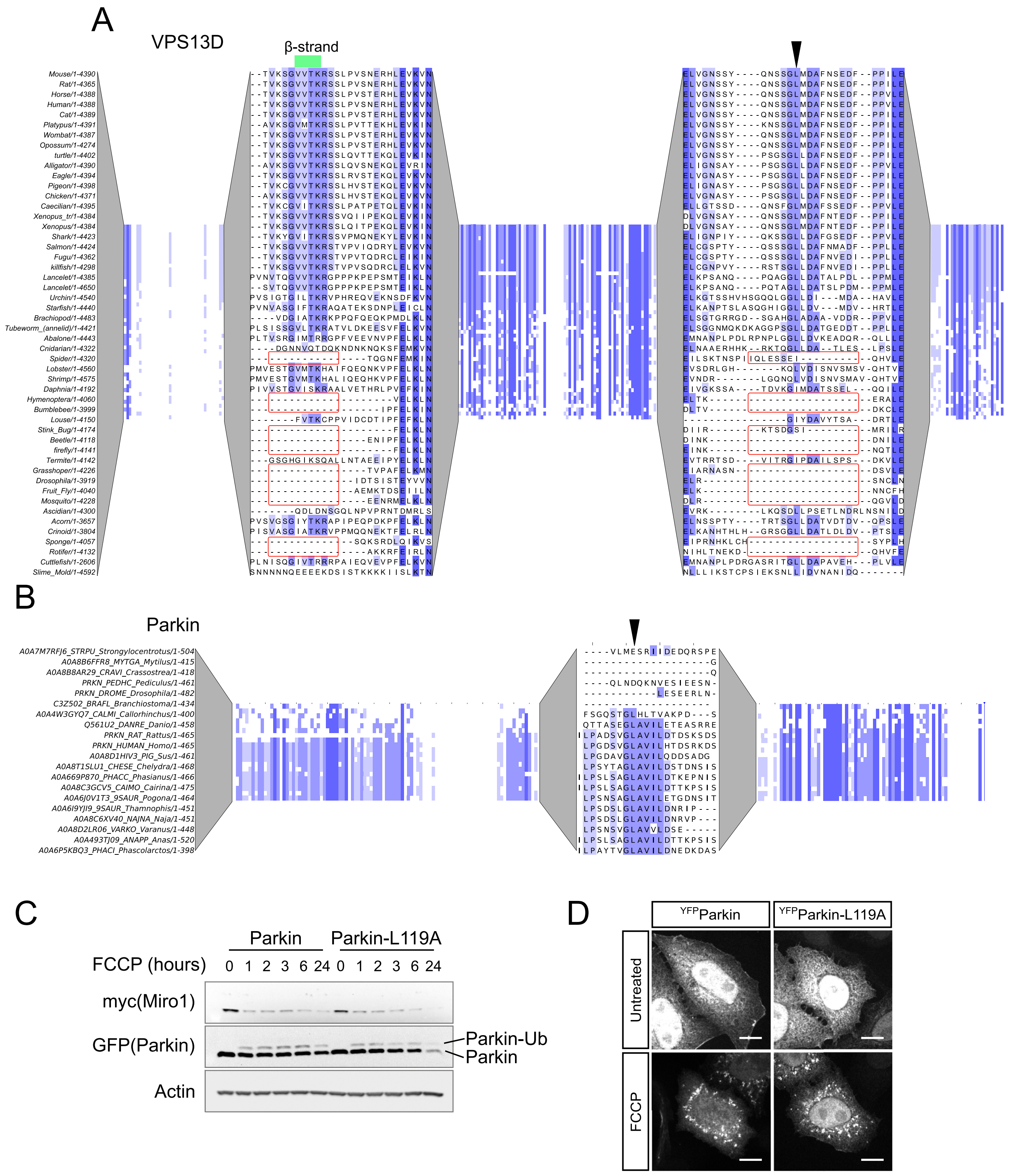
A
VPS13D
β-strand
B
Parkin
C
Parkin
Parkin-L119A
FCCP (hours)
0 1 2 3 6 24 0 1 2 3 6 24
myc(Miro1)
GFP(Parkin)
Parkin-Ub
Parkin
Actin
D
YFPParkin
YFPParkin-L119A
Untreated
FCCP

Figure EV5. Conservation of VPS13D and Parkin Miro-binding motifs.

(**A**) Sequence alignment of VPS13D. Red boxes show sequences that lack both the β-strand and the Leucine-containing motif of the MBM together. (**B**) Sequence alignments of Parkin. Arrowheads point to the conserved leucine residues mutated in this study. (**C**) HeLa cells transfected with WT or L119A mutant Parkin and treated with 10 μM FCCP to induce mitochondrial depolarization. Timepoints collected as indicated were analyzed by western blotting using the indicated antibodies. (**D**) YFP-Parkin signal in untreated and 10 μM FCCP treated (1 h) HeLa cells. Scale bar is 10 μm.

© The Author(s)
